# Systematic Screening of Chemical Constituents in the Traditional Chinese Medicine *Arnebiae Radix* by UHPLC-Q-Exactive Orbitrap Mass Spectrometry

**DOI:** 10.3390/molecules27092631

**Published:** 2022-04-19

**Authors:** Lian Zhu, Shengjun Ma, Kailin Li, Pei Xiong, Shihan Qin, Wei Cai

**Affiliations:** 1 College of Food Science and Pharmacy, Xinjiang Agricultural University, Urumqi 830052, China; zhu854422876@163.com (L.Z.); wlmqmsj@sina.com (S.M.); 2 Sino-Pakistan Center on Traditional Chinese Medicine, School of Pharmaceutical Sciences, Hunan University of Medicine, Huaihua 418000, China; lkl20182022@163.com (K.L.); zjtxzwxpxh@163.com (P.X.); ssd869132@163.com (S.Q.)

**Keywords:** *Arnebiae Radix*, identification, chemical constituents, UHPLC-Q-Exactive Orbitrap MS

## Abstract

*Arnebiae Radix* (dried root of *Arnebia euchroma* (Royle) Johnst.) has been used in traditional Chinese medicine (TCM) to treat macular eruptions, measles, sore throat, carbuncles, burns, skin ulcers, and inflammation. Previous studies have shown that shikonins and shikonofurans are two of their main bioactive ingredients. However, systematic investigations of their constituents have rarely been conducted. It is necessary to establish a rapid and effective method to identify the chemical constituents of *Arnebiae Radix*. This will help to further improve the effective resource utilization rate of this plant. In this study, a rapid and effective UHPLC-Q-Exactive Orbitrap mass spectrometry method was established to simultaneously analyze chemical ingredients in *Arnebiae Radix* within a short period of time. Based on the results of a full scan MS, the MS^2^ database (mzVault and mzCloud), the diagnostic fragment ions, the retention time, and the bibliography, a total of 188 compounds were identified, with 114 of those being reported from *Arnebiae Radix* for the first time. The results of this study lay the foundation for obtaining a thorough understanding of the active ingredients in *Arnebiae Radix* and its quality control. This method may be widely used for the chemical characterization of different samples.

## 1. Introduction

*Arnebiae Radix*, commonly called known as “Zicao” in traditional Chinese medicine (TCM), is the root of *Arnebia euchroma* (Royle) Johnst. It is primarily distributed in Mongolia, Xinjiang, and Northeast China [1,2]. It has been widely used as a folk medicine for clearing heat (Qingre) and for detoxification (Jiedu) by oral administration and for promoting blood circulation in local wounds via external application [3,4]. *Arnebiae Radix* has been used for many years for the treatment of macular eruptions, measles, sore throat, carbuncles, burns, skin ulcers, inflammation, allergic contact dermatitis(ACD) [5,6,7,8] and recently, for the treatment of cancer [9,10]. Previous studies have acknowledged the richness and complexity of its chemical composition. Its main active ingredients are the naphthoquinone compound, which has exhibited extensive antioxidant, antimicrobial, anti-inflammatory, and antitumor activities [11,12,13]. Many compounds have been reported to exist in *Arnebiae Radix*, including shikonins and shikonofurans [14,15]. However, the characterization of the constituents of *Arnebiae Radix* is still insufficient. Therefore, it is necessary to develop a systematic strategy for the rapid detection and identification of the constituents of *Arnebiae Radix*, as this will be very helpful for understanding its material basis and quality control.

The complexity of chemicals contained in TCM has presented a significant challenge regarding the rapid identification and characterization of components. Liquid chromatography-mass spectrometry (LC-MS), especially ultra-high performance liquid chromatography-high resolution mass spectrometry (UHPLC-HRMS), has been used extensively for qualitative analysis, quantitative analysis, and quality control of TCM due to its validity, sensitivity, and specificity [16,17]. HRMS provides high mass accuracy measurements for fragment ions with fast scan speeds. These features help with the identification of constituents with excellent accuracy and high reproducibility [18,19,20,21]. Cai et al. [22] used UHPLC-HRMS with parallel reaction monitoring (PRM) mode to unanimously and tentatively identify 149 chlorogenic acid derivatives from *D. nervosa,* which widely extended the knowledge on the chemical constituents of D. nervosa, facilitating the understanding of effective substances and quality control. Xiong et al. [23] used UHPLC-Q-Exactive Orbitrap mass spectrometry to systematically identify 106 constituent tannins in *Paeoniae Radix Alba* in negative ion mode. A systematic strategy was proposed for the rapid detection and identification of the chemical constituents of *Arnebiae Radix* using UHPLC-Q-Exactive Orbitrap mass spectrometry based on the expected compound and diagnosis fragmentation ion techniques.

The aim of the present investigation was to detect and identify the chemical constituents of *Arnebiae Radix* by UHPLC-Q-Exactive Orbitrap MS. In total, 188 compounds were identified in *Arnebiae Radix*, 114 of which are reported for the first time here. This result will improve the in-depth understanding of the pharmacological actions of *Arnebiae Radix* and lay a foundation for quality control of the drug for future clinical use.

## 2. Results and Discussion

### 2.1. Optimization of the UHPLC-Q-Exactive Orbitrap MS Condition

In order to acquire a better chromatographic peak shape and separation resolution, various factors were set to carry out a detection and identification process, including the column (Thermo Scientific Hypersil GOLD^TM^ aQ 100 mm × 2.1 mm, 1.9 μm and Waters ACQUITY BEH C18 column, 100 mm × 2.1 mm, 1.7 μm), column temperature (30, 35, 40 °C), and mobile phase gradient. The chemical constituents showed a high resolution and high sensitivity level based on the LC-MS conditions of “Section 3.3”.

### 2.2. Establishment of Diagnostic Fragment Ions (DFIs)

The DFI filter is a rapid screening method that is used to identify traditional Chinese medicine chemical components based on the accurate ion mass information provided by high-resolution mass spectrometry and the mass spectrometry fragmentation characteristics of similar chemical components. Thus, it is suitable for the identification of structural analogs. Different from a previous method of referring to the literature to establish a chemical database, the diagnostic fragment ions overcome the limitation of finding potential new compounds and deduce the overall structure of the compounds from the fragmentation information of the mass spectrum of the compound [24,25,26]. Generally, it is well-known that chemical constituents in the same category possess identical carbon skeletons and homologous biosynthetic pathways. It is easily understood that shikonin derivatives, phenolic acids, and flavonoids with the same carbon skeletons will generate similar fragmentation patterns, and these can be defined as DFIs for screening and characterization.

**Figure 1:**
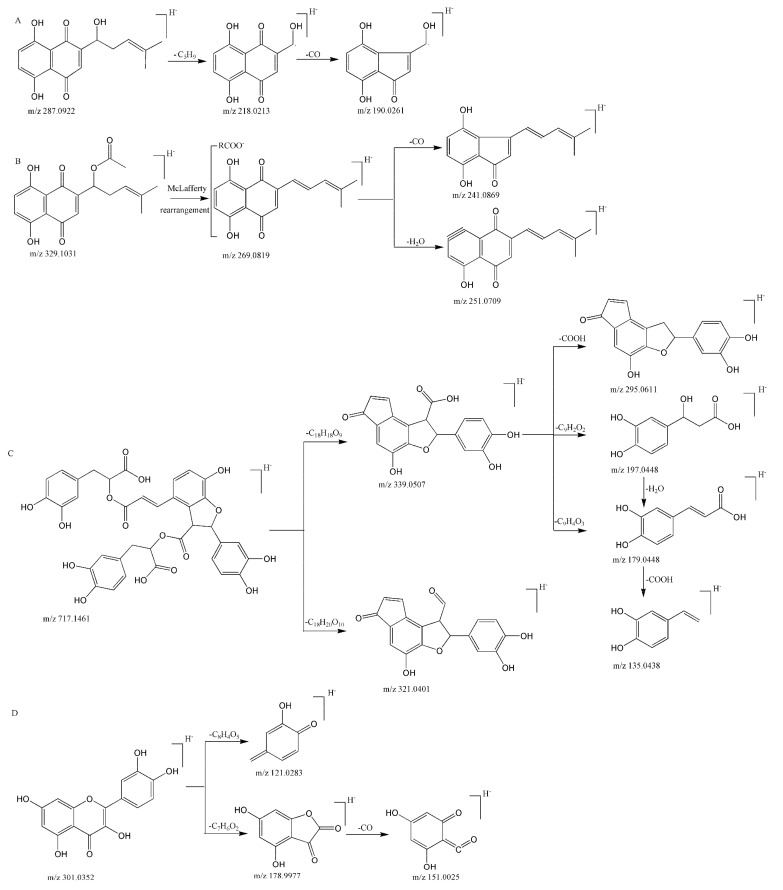
Fragmentation routes of the reference standards: shikonin (**A**); acetylshikonin (**B**); salvianolic acid B (**C**); quercetin (**D**).

In this study, the fragmentation patterns of 14 reference standards were investigated by UHPLC-Q-Exactive Orbitrap MS in negative mode to establish the DFIs, and the selected fragmentation patterns of the components are shown in Figure 1. In shikonin, the deprotonated molecular ion [M − H]^−^ produced *m*/*z* 287.0925 (C_16_H_15_O_5_) *m*/*z* 218.0213 and 190.0261 as the predominant fragment ions by the loss of C_5_H_9_ and CO, respectively. For acetylshikoninor β,β′-dimethylacrylalkannin, the deprotonated molecular ion [M − H]^−^ at *m*/*z* 329.1031 (C_18_H_17_O_6_) and 369.1344 (C_21_H_21_O_6_) produced *m*/*z* 269.0819, 251.0709 and 241.0869 as the predominant fragment ions.

The quasi-molecular ion of the reference standards salvianolic acid B is *m*/*z* 717.14610 in negative mode. The parent ion yielded the fragment ions *m*/*z* 339.0507 (C_18_H_11_O_7_) and 321.0401 (C_18_H_9_O_6_) by the loss of C_18_H_18_O_9_ and C_18_H_20_O_10_, respectively. With the breaking of the ester bond between the carbonyl group and the oxygen atom, 197.0446 (C_9_H_9_O_5_) was obtained. In addition, *m*/*z* 197.0446 (C_9_H_9_O_5_) produced the fragment ions *m*/*z* 179.0448 (C_9_H_7_O_4_) and 135.0439 (C_8_H_7_O_2_) by the loss of H_2_O and COOH. All of the above ions can be used as DFIs of phenolic acid.

Quercetin yielded a deprotonated molecular ion [M − H]^−^ at *m*/*z* 301.0359 (C_15_H_9_O_7_), which initially produced 151.0025 (C_7_H_6_O_2_) and 178.9977 (C_8_H_4_O_5_) by Retro Diels–Alder (RDA) rearrangement. The diagnostic ion 178.9977 was shown to eliminate a molecule of CO_2_ to yield relative fragment ions of 121.0283 (C_7_H_4_O_4_).

### 2.3. Characterization of the Chemical Constituents in Arnebiae Radix

**Figure 2:**
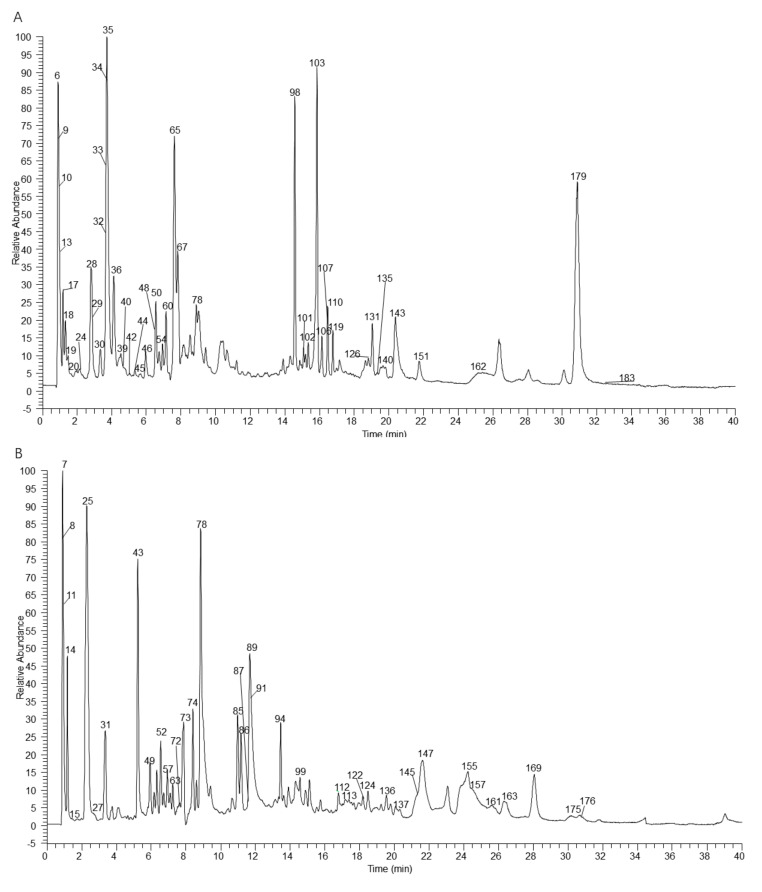

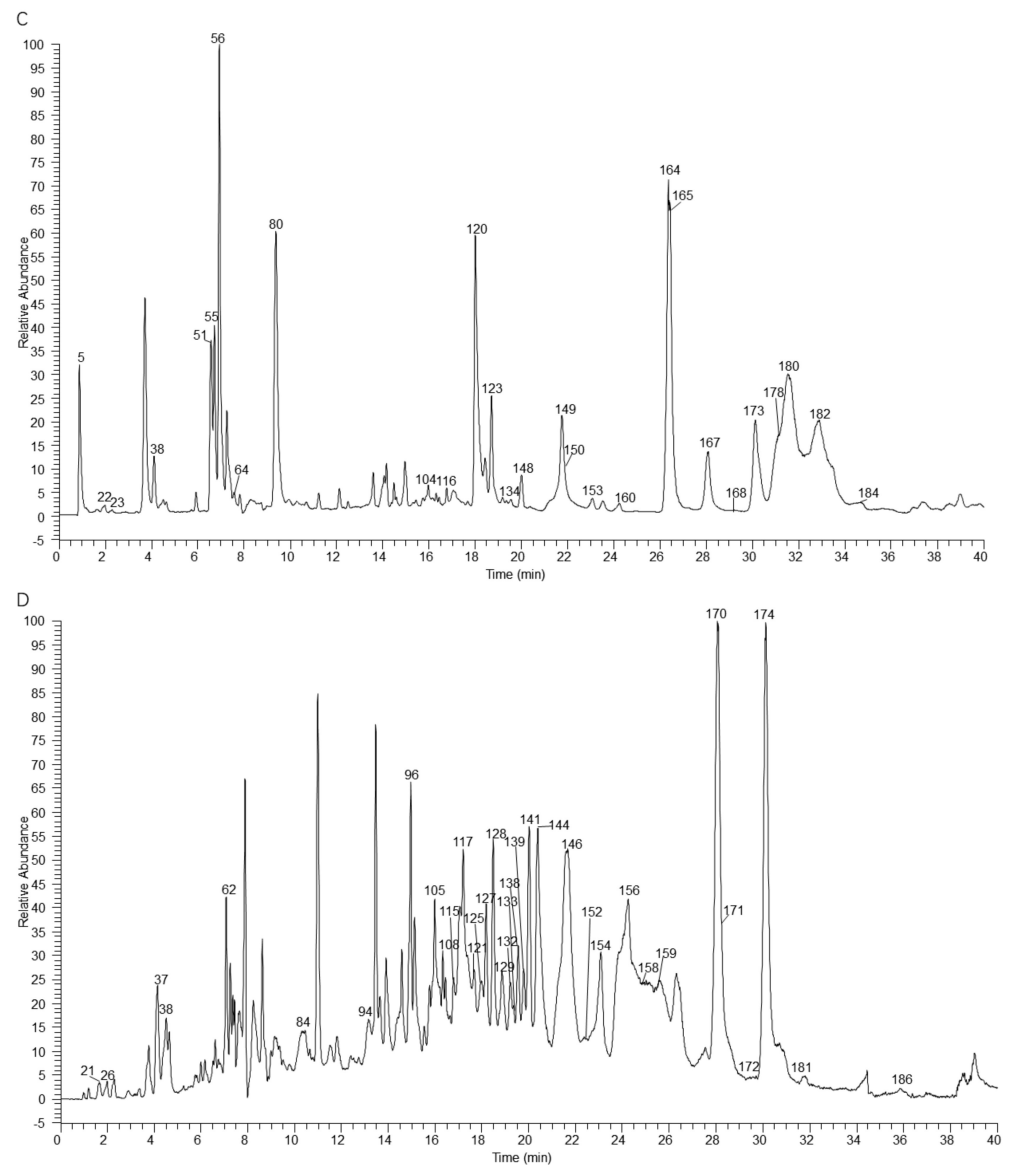

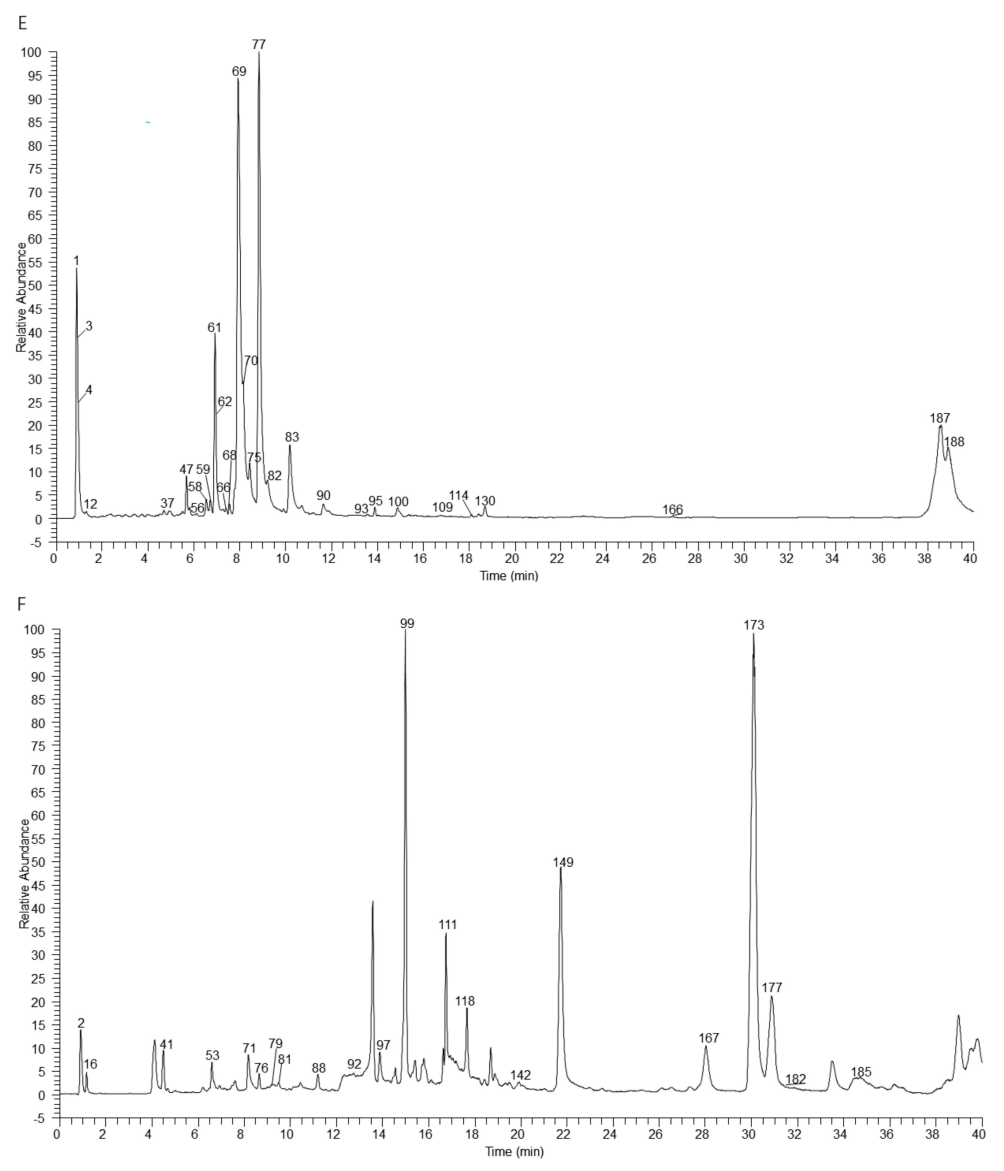
The high-resolution extracted ion chromatogram (HREIC) in 10 ppm for multiple compounds in *Arnebiae Radix*. (**A**) *m*/*z* 313.07176, 131.04621, 145.06186, 503.16175, 191.05611, 149.04554, 177.04046, 147.02989, 243.06225, 731.16175, 493.11402, 357.06159, 201.11323, 253.08701, 315.12379, 299.05611, 583.25620, 175.06119, 153.01933, 188.03531, 172.09791, 151.04006, 193.05063, 375.13100, 163.04006, 206.08226, 173.08193, 174.05605, 449.10893, 137.02441, 715.13045, 551.11949, 277.14453, 167.02106, 161.04554, 145.05063, 164.07170, 218.10339, 158.08226, 181.05063, 203.08260, 187.13396, 371.15001, 269.08193, 333.13436, 345.09797, 357.20713, 375.18131, 387.14492; (**B**) *m*/*z* 491.09837, 609.14610, 731.16175, 135.02989, 195.05102, 193.03537, 191.01972, 197.04554, 167.03498, 161.02441, 179.03498, 163.04006, 493.11402, 463.08819, 593.15119, 187.09758, 277.14453, 373.09289, 305.17583, 313.07176, 363.10854, 283.06119, 347.11362, 349.12927, 299.05611, 361.16566, 269.04554, 355.15509, 425.12419, 357.17074, 459.16605, 373.12927, 445.15040, 285.07684, 399.14492, 385.12927, 401.16057, 599.19227, 465.10384, 433.11402; (**C**) *m*/*z* 179.05611, 287.09249, 555.16605, 405.15549, 137.02441, 329.10306, 369.13436, 627.22357, 537.10384, 429.15549, 445.15040, 271.09758, 343.15509, 433.18679, 285.04046, 343.11871, 373.16566, 357.13436, 357.06159, 391.13984, 169.01424, 417.08271, 465.10384, 289.07176, 433.11402, 403.13984, 391.17622; (**D**) *m*/*z* 363.10854, 343.15509, 433.18679, 285.04046, 343.11871, 373.16566, 357.13436, 357.06159, 391.13984, 169.01424, 417.08271, 465.10384, 289.07176, 433.11402, 403.13984, 391.17622, 283.06119, 347.11362, 349.12927, 405.15549, 299.05611, 361.16566, 269.04554, 355.15509, 359.11362, 425.12419, 357.17074, 387.14492, 459.16605, 373.12927, 445.15040, 285.07684, 399.14492, 385.12927, 401.16057, 369.13436, 599.19227, 627.22357; (**E**) *m*/*z* 131.04621, 465.10384, 433.11402, 449.10893, 463.08819, 493.11402, 551.11949, 731.16175, 271.06119, 583.25620, 283.06119, 357.20713, 537.10384, 133.01424, 429.15549, 341.10893, 387.14492, 359.11362, 387.11441, 639.22357, 329.10306, 717.14610,359.07724; (**F**) *m*/*z* 609.18249, 301.03537, 357.17074, 271.09758, 369.13436, 313.07176, 131.04621, 145.06186, 503.16175, 191.05611, 149.04554, 177.04046, 147.02989, 243.06225, 731.16175, 493.11402, 357.06159, 201.11323, 253.08701, 315.12379, 299.05611 ((**A**–**D**,**E**,**F**) correspond to the EIC of Samples B, C, A, respectively).

The table lists all the chemical constituents detected in the extracted *Arnebiae Radix* sample by UHPLC-Q Exactive Orbitrap mass spectrometry based on the diagnostic fragment ions, retention time, MS^2^ database (mzVault and mzCloud), and bibliographical identification (Table 1). A total of 188 chemical constituents (114 first report) were accurately or tentatively identified. The extracted ion chromatogram (EIC) in negative ion mode was obtained, as shown in Figure 2. Large differences in chemical constituents were seen in the different batches of *Arnebiae Radix* samples (A, B, and C), indicating that the chemical constituents of *Arnebiae Radix* differ significantly in the current Chinese medicine market, possibly due to the different growth environments, growth periods, and medicinal material storage times used [27]. Generally, the samples from batch C from southern Xinjiang showed obvious advantages in terms of their peak intensity and number of peaks.

#### 2.3.1. Identification of Shikonins

**Figure 3:**
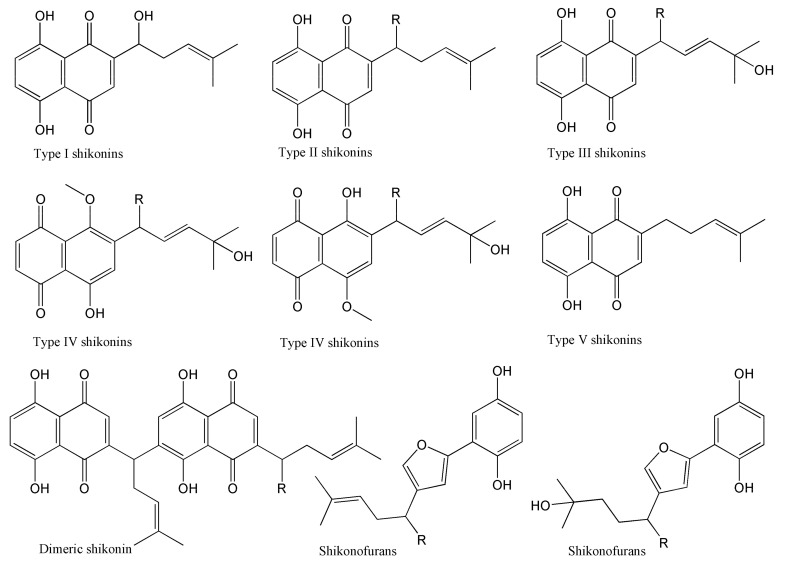
Chemical structures of 7 different types of shikonin compounds found in *Arnebiae Radix*.

It is well-known that the common structural features of most shikonins are a skeleton of naphthoquinone combined with an isohexenyl side chain. Based on a comparison with fragment ions detailed in the literature and reference standards, this study identified seven different types of shikonin compounds in the crude extract, and the chemical structures of these are shown in Figure 3.

##### Type I Shikonins

Shikonins, composed of a naphthoquinone moiety and an isohexenyl side chain, were identified based on the DFIs at *m*/*z* 218.0213 and 190.0261. In total, five type I shikonins (94, 103, 105, 116, and 120) were unambiguously or tentatively identified. For instance, shikonin (120, R.T. = 18.04 min) was unambiguously identified by comparing the listed diagnostic ions and retention times with reference standards. Compounds **94**, **103**, and **105** were found at 13.13, 15.85, and 15.99 min, and these generated similar fragment ions as the shikonins. Thus, they were tentatively characterized as shikonin derivatives. Compound **116**, which was observed at 17.00 min yielded a deprotonated ion [M − H]^−^ *m*/*z* 405.15549 and was tentatively identified as dihydrohydroxyshikonin tiglate [28].

##### Type II Shikonins

Compounds **150** and **174** found at 21.89 and 30.19 min, yielded deprotonated ions [M − H]^−^ *m*/*z* 329.10306 (C_18_H_17_O_6,_ −0.19 ppm) and 369.13436 (C_21_H_21_O_6,_ −0.39 ppm), respectively. Through a comparison with the reference standards, they were identified as acetylshikonin and β,β′-dimethylacrylshikonin. Compounds **102**, **143**, **179**, and **183**, which were found at 15.74, 20.16, 30.98, and 33.05 min, showed a common precursor ion at [M − H]^−^ *m*/*z* 371.1500 (C_21_H_24_O_6_). They mainly yielded fragment ions at *m*/*z* 269.0819, 241.0869, and 251.0709 and were tentatively characterized as α,α-dimethylpropionylshikonin, α-methylbutyrylshikonin, isovalerylshikonin, and isovalerylshikonin isomer through a comparison with the literature and MS^2^ fragmentation [27,28]. Compound **144**, found at 20.50 min, showed a precursor ion at [M − H]^−^ *m*/*z* 387.14492. Therefore, it was tentatively characterized as β-hydroxyisovalerylshikonin [27,28]. Compounds **166** and **168** appeared at retention times (tR) of 26.60 min and 28.07 min, respectively. The parent ion *m*/*z* 429.15549 generated the characteristic fragments *m*/*z* 269.0822, 251.0709, and 241.0869, which were tentatively identified as 5-acetoxy-valerylshikonin and β-acetoxyisovalerylshikonin [27]. Similarly, compounds **170** and **171** were tentatively characterized as butyrylshikonin and isobutyrylshikonin. Overall, compounds **177**, **182**, and **185** demonstrated highly similar product ion spectra and MS/MS fragmentation behaviors but had different retention times, suggesting that they are isobaric compounds. Therefore, they were tentatively identified as α-methylene-butenoylshikonin, tigloylshikonin, and angeloylshikonin, as previously reported [27].

##### Type III Shikonins

Due to the presence of a double bond between C-12 and C-13, the α-cleavage mechanism of the ester carbonyl is the main fragmentation pathway of the type III shikonins producing product ion at *m*/*z* 285.0766. This ion formed the diagnostic generation product ions 257.0807 and 267.0674 by successive neutral loss of H_2_O and CO, respectively [27]. Compounds **146** and **162** had the same molecular formula C_21_H_24_O_7_. In addition, diagnostic products at *m*/*z* 285.0766, 267.0663, and 257.0815 were observed through MS^2^ fragmentation ion. Based on a literature search of all shikonins reported [29], compounds **146** and **162** were tentatively identified as lithospermidin A and lithospermidin B, respectively. The ESI-MS of compounds **119**, **148**, **152**, and **158** gave deprotonated molecular ions at *m*/*z* 345.09797, 445.15040, 373.12927, and 385.12927, which matched the molecular formulas C_18_H_18_O_7_, C_23_H_26_O_9_, C_20_H_22_O_7__,_ and C_21_H_22_O_7_, respectively. Consequently, these compounds were tentatively identified as lithospermidin C, lithospermidin D, lithospermidin E, and lithospermidin F [27]. Compound **126** was eluted at 18.65 min and generated the same MS and MS^2^ fragmentation results as compound **119**. Thus, this compound was tentatively identified as the lithospermidin C isomer. Compounds **153**, **156**, and **164** appeared at retention times (tR) of 23.03 min, 24.23 min, and 26.38 min, respectively. They had the same MS and MS^2^ fragmentation ions as compound **148** and were tentatively identified as lithospermidin D isomers.

##### Type IV Shikonins

Diagnostic ions were identified at *m*/*z* 299.0921, 284.0687, and 271.0973 for type IV shikonins, and fragmentation ions were obtained at *m*/*z* 284.0687 and at *m*/*z* 271.0973 via the neutral loss of CH_3_ and CO from 299.0921, respectively [27]. As an example, compound 138 with a retention time at 19.74 min showed a deprotonated molecular ion at *m*/*z* 359.11362 (C_19_H_19_O_7_, 0.82 ppm) by ESI-MS. Diagnostic product ions were located at 299.0921, 284.0697, and 271.0973 in MS^2^ and the compound was tentatively identified as 1/4-methoxylithospermidin C [27,28]. According to the literature [29,30] and the different retention times, isomers in each group were eluted based on a predefined order. Compounds **147**, **151**, **154**, **157**, **159**, **161**, **163**, **172**, and **175** were predicted to be 1/4-methoxylithospermidin H, 1/4-methoxylithospermidin I, 1/4-methoxylithospermidin E, 1/4-methoxylithospermidin J, 1/4-methoxylithospermidin F, 1/4-methoxylithospermidin A, 1/4-methoxylithospermidin D, 1/4-methoxylithospermidin L, and 1/4-methoxylithospermidin B from the experimental chromatographic peaks [27,28]. By comparing MS and MS^2^ fragmentation in the literature, compound **145** was tentatively assigned as 1-methoxy-β-hydroxyisovalerylshikonin [28].

##### Type V Shikonins

Deoxyshikonin, a precursor for the biosynthesis of other shikonins, was not classified as any type of shikonin or shikonofuran due to the absence of a carboxylic group. The deprotonated molecular ion at *m*/*z* 271.0968 for deoxyshikonin gave a product ion base peak at *m*/*z* 203.0342. In addition, a product ion at *m*/*z* 253.0867 was derived from the neutral loss of H_2_O from the deprotonated molecular ion [27]. Compounds **149**, **165**, **167**, **173**, **178**, and **184** were eluted at 21.76, 26.38, 28.04, 30.11, 30.89, and 34.64 min and showed a deprotonated molecular ion [M − H]^−^ at *m*/*z* 271.098 (C_16_H_15_O_4_). According to a previously published paper [27,28] and the MS^2^ fragmentation results, these compounds were tentatively assigned as deoxyshikonin or its isomers based on the different base peak ions in the MS^2^ spectrum.

##### Shikonofurans

According to previous studies [27], *m*/*z* 255.1024 is the DFIs of shikonofurans, and it generates fragment ions at *m*/*z* 237.0917 and *m*/*z* 227.0340 through the neutral loss of H_2_O and CO, respectively. The compounds **111**, **127**, **136**, **141**, and **142** were eluted at 16.76, 18.83, 19.52, 20.06, and 20.15 min and showed deprotonated molecular ions [M − H]^−^ at *m*/*z* 315.12379 (−0.06 ppm, C_18_H_19_O_5_), 343.15509 (−0.03 ppm, C_20_H_23_O_5_), 355.15509 (−1.49 ppm, C_21_H_23_O_5_), 357.17074 (−0.61 ppm, C_37_H_35_O_10_), and 357.17074 (−0.95 ppm, C_37_H_35_O_10_), suggesting that they are shikonofurans. Therefore, compounds **111**, **127**, **136**, **141**, and **142** were identified as shikonofuran A, shikonofuran D, shikonofuran E, shikonofuran B, and shikonofuran C, respectively, by comparing their respective MS^2^ data and data presented in the literature [27]. According to the DFIs, compound **115** was tentatively identified as a shikonofuran derivative. In addition, DFIs at *m*/*z* 273.1133 were used for the classification of another shikonofuran indicating that the hydration was likely on the double bond of the side chain. A peak at *m*/*z* 255.1024 was observed due to neutral losses of H_2_O from DFI 273.1133 [27]. Compounds **104**, **107**, **108**, **110**, **121**, **122**, **123**, **124**, **129**, **132**, **133**, **135**, **137** and **140** eluted at 15.96, 16.13, 16.31, 16.46, 18.15, 18.17, 18.45, 18.47, 18.92, 19.16, 19.33, 19.49, 19.67, and 19.80 were predicted to be hydroxyshikonofurans due to the fragment ions yielded at *m*/*z* 273.1133 and *m*/*z* 255.1024. Therefore, they were tentatively identified as hydroxyshikonofuran J, hydroxyshikonofuran A, hydroxyshikonofuran K, hydroxyshikonofuran A isomer, hydroxyshikonofuran F, hydroxyshikonofuran G, hydroxyshikonofuran H, hydroxyshikonofuran D, hydroxyshikonofuran I, hydroxyshikonofuran E, hydroxyshikonofuran B, hydroxyshikonofuran L, hydroxyshikonofuran C, and hydroxyshikonofuran M [27,28]. Compounds **128** and **134** were found at 18.89 and 19.33 min and showed a common precursor ion at [M − H]^−^ *m*/*z* 343.11871. They were tentatively characterized as 1-methoxyacetylshikonin and its isomer, respectively [28].

##### Dimeric Shikonin

Compounds **176**, **181**, **186**, **187**, and **188** were eluted at 30.64, 31.78, 35.84, 38.59, and 38.89 min and showed deprotonated molecular ions [M − H]^−^ at *m*/*z* 599.19227 (C_34_H_31_O_10_), 599.19227 (C_34_H_31_O_10_), 627.22357 (C_36_H_35_O_10_), 639.22357 (C_37_H_35_O_10_), and 639.22357 (C_37_H_35_O_10_). According to a previously published paper [28], they were tentatively assigned as 7-(11′-deoxyalkannin)-acetylshikonin,7-(11′-deoxyalkannin)-acetylalkannin, 7-(11′-deoxyalkannin)-isobutyrylshikonin, 7-(11′-deoxyalkannin)-β,β-dimethylacrylshikonin, and 7-(11′-deoxyalkannin)-β,β-dimethylacrylalkannin based on the different base peak ions in the MS^2^ spectrum listed in Table 1.

#### 2.3.2. Identification of Phenolic Acids

Four compounds—rosmarinicacid (**77**), lithospermic acid (**80**), salvianolic acid B (**83**), and salvianolic acid C (**89**)—were unambiguously identified by comparing their MS and MS^2^ fragmentation ion and retention times with the reference standards in negative ionization mode.

Compound **69** produced the same MS and MS^2^ fragmentation results as compound **83** (salvianolic acid B). Thus, this compound was tentatively identified as salvianolic acid B isomer. Compounds **51**, **55**, **56**, and **70** were found at 6.54, 6.71, 6.91, and 8.02 min and showed a common precursor ion at [M − H]^−^ at *m*/*z* 537.1039 (C_27_H_21_O_12_). They yielded *m*/*z* 197.0446 by the breaking of the ester bond between the carbonyl group and the oxygen atom, *m*/*z* 179.0448 (C_9_H_7_O_4_) due to the loss of H_2_O units, and *m*/*z* 135.0439 (C_8_H_7_O_2_) by the loss of COOH. According to a previously published paper [31], they were tentatively assigned as salvianolic acid U, salvianolic acid T, salvianolic acid J, and salvianolic acid isomer based on the different base peak ions in the MS^2^ spectrum. Compounds **25**, **26**, **43**, **65**, **71**, **84**, **87**, and **130** were eluted at 2.28, 2.28, 5.22, 7.60, 8.19, 10.33, 11.16, and 18.99 min with deprotonated ions [M − H]^−^ at *m*/*z* 197.04554 (C_9_H_9_O_5_), 417.08271 (C_20_H_17_O_10_), 179.03498 (C_9_H_7_O_4_), 359.07724 (C_18_H_15_O_8_), 357.06159 (C_18_H_13_O_8_), 357.06159 (C_18_H_13_O_8_), 373.09289 (C_19_H_17_O_8_), and 357.20713 (C_22_H_29_O_4_). They were tentatively identified as danshensu, salvianolic acid D, caffeic acid, didehydiosalvianolic acid B, methyl rosmarinate, and cannabidiolic acid [31,32,33,34,35]. Likewise, compounds **53** and **57**; compounds **58**, **81**, and **82**; compounds **68**, **75**, and **90**; and compounds **78**, **79**, **85**, **91**, and **93** produced similar diagnostic ions and MS^2^ fragmentation behaviors. They were provisional identified as salvianolic acid F, salvianolic acid A, monomethyl lithospermate, 9″-methyl salvianolate B, and their isomers, respectively.

Compound **3** was eluted at 0.88 min with deprotonated ions [M − H]^−^ at *m*/*z* 387.11441 (−1.44 ppm, C_13_H_23_O_13_), and the presence of fragment ions at *m*/*z* 341.1084 and 179.0550 was used to tentatively identify it as 2,3,4,5,6-pentahydroxy-7-[(2*S*,3*R*,4*S*,5*S*,6*R*)-3,4,5-trihydroxy-6-(hydroxymethyl)oxan-2-yl]oxyheptanoic acid [28]. Compounds **4**, **5**, and **6** were eluted at the same time (0.88 min) and produced the same fragment ions (89.0230, 101.0230, 113.0231), which were initially identified as α, α-trehalose, mannose, and raffinose. Compound **10** was eluted at 0.91 min with deprotonated ions [M − H]^−^ at *m*/*z* 149.04554 and was initially identified as arabinose. By searching in the databases (ChemSpider, mzVault, and mzCloud), compounds **7**, **8**, **11**, **12**, **13**, **16**, **19**, and **101** were eluted at 0.89, 0.90, 0.92, 0.93, 0.93, 1.17, 1.31, and 15.35 min with deprotonated ions [M − H]^−^ at *m*/*z* 135.02989 (C_4_H_7_O_5_), 195.05102 (C_6_H_11_O_7_), 193.03537(C_6_H_9_O_7_), 133.01424 (C_4_H_5_O_5_), 177.04046 (C_6_H_9_O_6_), 147.02989(C_5_H_7_O_5_), 161.04554(C_6_H_9_O_5_), and 187.13396 (C_10_H_19_O_3_), respectively. They were tentatively identified as threonic acid, gluconic acid, β-D-glucopyranuronic acid, malic acid, δ-gluconic acid δ-lactone, α-hydroxyglutaric acid, 3-hydroxy-3-methylglutaric acid, and 3-hydroxydecanoic acid. According to the literature [36,37], compounds **9**, **14**, and **15**, which were eluted at 0.90, 0.94, and 1.17 min were identified as quinic acid, citric acid isomer, and citric acid. Based on the literature [28,31,32,33,34,35,36,37,38] and a database search (ChemSpider, mzVault and mzCloud), compounds **18**, **20**, **21**, **23**, **24**, **27**, **28**, **31**, **33**, **35**, **36**, **39**, **40**, **44**, **45**, **49**, **50**, **52**, **54**, **60**, **64**, **67**, **74**, **88**, and **113** were tentatively assigned as uric acid, 3-methylglutaric acid, gallic acid, salicylic acid, pantothenic acid, vanillic acid, protocatechuic acid, isovanillic acid, 2-isopropylmalic acid, gentisic acid, kynurenic acid, 3-coumaric acid, p-coumaric acid, 2-hydroxyphenylacetic acid, ferulic acid, 4-methoxysalicylic acid, suberic acid, 4-coumaric acid isomer, indole-3-acetic acid, isoferulic acid, salicylic acid isomer, 4-coumaric acid, azelaic acid, 3-tert-butyladipic acid, and octyl ferulate based on the different base peak ions in the MS^2^ spectrum listed in Table 1.

#### 2.3.3. Identification of Flavonoids

A total of 24 individual flavonoid constituents were putatively identified in *Arnebiae Radix* extract using UHPLC-Q-Exactive Orbitrap MS. Eight compounds, including rutin (**63**), isoquercitrin (**66**), nicotiflorin (**73**), hesperidin (**76**), quercetin (**92**), naringenin (**95**), kaempferol (**96**), and baicalein (**114**) were identified as having a pseudomolecular ion [M − H]^−^ at *m*/*z* 609.14610 (−0.79 ppm, C_27_H_29_O_16_), 463.08819 (−0.28 ppm, C_21_H_19_O_12_), 593.15119 (−0.43 ppm, C_27_H_29_O_15_), 609.18249 (1.61 ppm, C_28_H_33_O_15_), 301.03537 (−0.22 ppm, C_15_H_9_O_7_), 271.06119 (0.31 ppm, C_15_H_11_O_5_), 285.04046 (1.30 ppm, C_15_H_9_O_6_), and 283.06119 (−0.03 ppm, C_16_H_11_O_5_), respectively. These were unambiguously identified by comparing their accurate mass information and chromatography retention times with reference standards in negative ionization mode. Compounds **37**, **41**, **47**, **59**, **61**, **62**, and **72** yielded deprotonated molecular ions [M − H]^−^ at *m*/*z* 465.10384 (−0.20 ppm, C_21_H_21_O_12_), 465.10384 (−0.26 ppm, C_21_H_21_O_12_), 449.10893 (0.17 ppm, C_21_H_21_O_11_), 433.11402 (−0.17 ppm, C_21_H_21_O_10_), 449.10893 (0.17 ppm, C_21_H_21_O_11_), 433.11402 (−0.17 ppm, C_21_H_21_O_10_) and 277.14453 (−0.99 ppm, C_16_H_21_O_4_), which initially produced 151.0025 (C_7_H_6_O_2_), and 178.9977 (C_8_H_4_O_5_) by Retro Diels–Alder (RDA) rearrangement. According to a previous study [39,40] and the DFIs, they were tentatively characterized as taxifolin-glucoside, taxifolin-glucoside, eriodictyol-glucoside, naringenin-glucoside, eriodictyol-glucoside, naringenin-glucoside and De-O-methyllasiodiplodin. Compounds **99**, **100**, **109**, **118**, **125**, and **131** were tentatively assigned as hispidulin, bilirubin, glycitein, pectolinarigenin, apigenin, and medicarpin by searching in the databases such as the chemical structure database (ChemSpider) and MS^2^ database (mzVault and mzCloud). Peaks **38**, **46**, and **117** were eluted at 4.62, 5.94, and 19.11 min with deprotonated ions [M − H]^−^ at *m*/*z* 289.07176 (C_15_H_13_O_6_), 375.13100 (C_17_H_19_N_4_O_6_), and 345.09797 (C_18_H_17_O_7_). They were tentatively characterized as catechin, riboflavin, and kaempferide [39,40,41,42,43].

#### 2.3.4. Identification of Amino Acids

Compounds **1**, **2**, **22**, **29**, **32**, **42**, and **48** yielded a quasi-molecular ions [M − H]^−^ at *m*/*z* 131.04621 (C_4_H_7_N_2_O_3_), 145.06186 (C_5_H_9_N_2_O_3_), 164.07170 (C_9_H_10_NO_2_), 158.08226 (C_7_H_12_NO_3_), 203.08260 (C_11_H_11_N_2_O_2_), 172.09791 (C_8_H_14_NO_3_), and 206.08226 (C_11_H_12_NO_3_) and were eluted at 0.83, 0.86, 1.92, 2.96, 3.46, 5.17, and 6.01 min, respectively. Comparing the MS^2^ fragment ions with data from the bibliography [37,44,45], compounds **1**, **2**, **22**, **29**, **32**, **42**, and **48** were tentatively identified as asparagine, glutamine, phenylalanine, N-acetylvaline, tryptophan, N-acetyl-L-leucine, and N-acetyl-L-phenylalanine, respectively.

#### 2.3.5. Others

Compounds **7**, **30**, **34**, and **86** were tentatively identified as threonic acid, ethyl 3,4-dihydroxybenzoate, 7-hydroxycoumarin, and dibutylphthalate by searching in databases such as the chemical structure database (ChemSpider) and MS^2^ database (mzVault and mzCloud).

Compound **97**, found at 14.56 min, possessed a quasi-molecular ion [M − H]^−^ at *m*/*z* 253.08701 and was tentatively identified as Rhizonone [28].

## 3. Materials and Methods

### 3.1. Chemicals and Reagents

MS grade formic acid and acetonitrile were purchased from Thermo Fisher Scientific Co., Ltd. (Waltham, MA, USA). Ultra-pure water was obtained from Guangzhou Watsons Food & Beverage Co., Ltd. (Guangzhou, China). Other solvents were of analytical grade and were supplied by the Aladdin Industrial Corporation (Shanghai, China). The chemical reference standards of shikonin, acetylshikonin, β,β-dimethylacrylshikonin, lithospermic acid, rosmarinic acid, salvianolic acid B, and salvianolic acid C were purchased from Chengdu Pufei De Biotech Co., Ltd. (Chengdu, China), while those of nicotiflorin, rutin, isoquercitrin, quercetin, hesperidin, naringenin, kaempferol, and baicalein were provided by Cheng Du Herbpurify Co., Ltd. (Chengdu, China). The purities of all reference standards were above 98% according to an HPLC-UV analysis. The brand and model of the sonication device was KQ-300DE from Kunshan Ultrasonic Instrument Co., Ltd. (Kunshan China).

### 3.2. Sample and Standard Preparation

A total of three batches of *Arnebiae Radix* were used in this study. Batch A samples were collected from Bortala Mongol Autonomous Prefecture, Xinjiang Uygur Autonomous Region, China; batch B samples (1703060112) were purchased from the herbal medicine markets; and batch C samples were obtained from Zhaosu County and authenticated by Professor Shengjun Ma. The voucher specimens were deposited in the School of Pharmaceutical Sciences, Hunan University of Medicine.

*Arnebiae Radix* herbs were ground into powder before sample preparation and sieved through No. 40 mesh. Dried *Arnebiae Radix* powder (10 g) was sonication-extracted (300 W, 40 KHz) in 200 mL of methanol for 1 h at room temperature (16–24 °C), and then the extracted solution was filtered and dried by rotary evaporation. The extracts of these samples were redissolved and centrifuged at 12,000 rpm for 10 min to obtain the supernatant. A volume of 2 μL of supernatant was injected into UHPLC-Q-Exactive Orbitrap MS for analysis.

Standard solutions were prepared in methanol at a concentration of 1.00 mg/mL. The stock solutions of the reference standards were further diluted to obtain working solutions, and then these solutions were stored at 4 °C before analysis.

### 3.3. Instruments and Conditions

In order to acquire a better chromatographic peak shape and separation resolution, various factors were set in the detection and identification process, including a column (Thermo Scientific Hypersil GOLDTM aQ 100 mm × 2.1 mm, 1.9 μm and Waters ACQUITY BEH C18 column, 100 mm × 2.1 mm, 1.7 μm), column temperature (30, 35, 40 °C), and the mobile phase gradient.

Each LC-MS analysis was exercised on a Q-Exactive Focus Orbitrap MS connected to a Thermo Scientific Dionex Ultimate 3000 RS through an ESI source. Chromatographic separation was performed at 35 °C using a Thermo Scientific Hypersil GOLD^TM^ aQ (100 mm × 2.1 mm, 1.9 μm). The mobile phase was composed of 0.1% formic acid (A) and acetonitrile (B), and the flow rate was 0.3 mL/min. The following gradient was used: 0–2 min, 95–90% A; 2–5 min, 90–80% A; 5–10 min, 80–75% A;10–15 min, 75–50% A; 15–25 min, 50–45% A; 25–40 min, 45–20% A; 40–45 min, 20–5% A; 45–45.1 min, 5–95% A; 45–50 min, 95% A.

All samples were analyzed in negative mode using the following tuning method. In terms of the mass spectrometry conditions, the spray voltage was 3.2 kV, the sheath gas and auxiliary gas operated at flow rates of 35 arb and 10 arb, respectively the temperature of the capillary was 320 °C and that of the auxiliary gas heater was 350 °C, and the S-lens RF level is 60. In the mass range of *m*/*z* 100–1200, a high-resolution mass spectrum was obtained at a resolution of 70,000, which was detected by the Orbitrap analyzer. MS^2^ data at a resolution of 17,500 were obtained by data-dependent MS^2^ scanning or parallel reaction monitoring (PRM) mode. Nitrogen (purity ≥ 99.999%) served as the collision gas, which generated the fragment ions, and the energy level was set as a normalized collision energy of 30%.

### 3.4. Data Processing and Analysis

Xcalibur software version 4.2 (Thermo Fisher Scientific, San Jose, CA, USA) was used to obtain all high-resolution data including the full-scan MS and MS^2^ data. Peaks detected with intensities over 10,000 were selected for identification. The chemical formulas for all parent and fragment ions of the selected peaks were calculated from the accurate mass using a formula predictor by setting the parameters as follows: C [0–60], H [0–120], O [0–60], and N [0–10]. The mass tolerance of MS and MS^2^ was within 10 ppm.

## 4. Conclusions

In this research, an efficient strategy based on UHPLC Q-Exactive Orbitrap MS in negative ion mode was established to detect chemical components in *Arnebiae Radix*. A total of 188 constituents were identified, of which 114 were reported in *Arnebiae Radix* for the first time here, including shikonins, phenolic acids, and flavonoids. These were detected and identified based on their chromatographic retention, MS and MS^2^, and bibliography data. These results are very useful references for understanding the bioactive compounds of *Arnebiae Radix* and their utilization. Overall, the results lay the foundation for in-depth research on the pharmacodynamic material basis of *Arnebiae Radix*.

## Figures and Tables

**Table 1:** Chromatographic and mass data of the components detected in *Arnebiae Radix* though UHPLC-Q-Exactive Orbitrap MS.

No.	Batch	t_R_	Theoretical Mass *m*/*z*	Experimental Mass *m*/*z*	Error (ppm)	Formula	MS/MS Fragment (-)	Identification
1	A, B, C	0.83 ^#^	131.0462	131.0450	−9.05	C_4_H_8_N_2_O_3_	MS^2^[131]: 114.0183(100), 113.0343(65), 70.0284(37), 95.0237(25), 131.0449(22)	Asparagine
2	A, B, C	0.86 ^#^	145.0619	145.0606	−8.80	C_5_H_10_N_2_O_3_	MS^2^[145]: 127.0500(100), 128.0340(73), 145.0606(97), 102.0546(48), 109.0394(40)	Glutamine
3	A, B, C	0.88	387.1144	387.1139	−1.44	C_13_H_24_O_13_	MS^2^[387]: 89.0229(100), 119.0336(45), 179.0550(42), 341.1084(33), 161.0444(16), 221.0658(5)	2,3,4,5,6-pentahydroxy-7-[(2*S*,3*R*,4*S*,5*S*,6*R*)-3,4,5-trihydroxy-6-(hydroxymethyl)oxan-2-yl]oxyheptanoic acid
4	A, B, C	0.88 ^#^	341.1089	341.1084	−1.66	C_12_H_22_O_11_	MS^2^[341]: 89.0230(100), 59.0124(48), 71.0124(40), 101.0230(33), 119.0336(29), 113.0231(25)	α,α-Trehalose
5	A, B, C	0.88 ^#^	179.0561	179.0550	−6.27	C_6_H_12_O_6_	MS^2^[341]: 59.0124(100), 89.0230(68), 71.0124(63), 75.0073(48), 101.0230(33), 119.0336(29)	Mannose
6	A, B, C	0.88 ^#^	503.1618	503.1612	−1.19	C_18_H_32_O_16_	MS^2^[503]: 89.0230(100), 101.0230(50), 113.0230(38), 179.0551(26)	Raffinose
7	A, B, C	0.89 ^#^	135.0299	135.0286	−9.68	C_4_H_8_O_5_	MS^2^[135]: 75.0073(100), 135.0287(51), 72.9917(13), 89.0230(11), 59.0124(10)	Threonic acid
8	A, B, C	0.90 ^#^	195.0510	195.0500	−5.26	C_6_H_12_O_7_	MS^2^[195]: 195.0501(100), 75.0073(82), 129.0180(76), 99.0074(22), 87.0073(21), 59.0124(12)	Gluconic acid
9	A, B, C	0.90 ^#^	191.0561	191.0551	−5.25	C_7_H_12_O_6_	MS^2^[191]: 111.0074(100), 87.0073(35), 85.0280(28), 191.0551(12)	Quinic acid
10	A, B, C	0.91 ^#^	149.0455	149.0443	−8.17	C_5_H_10_O_5_	MS^2^[149]: 149.0443(100), 89.0230(76), 59.0124(32), 75.0073(32)	Arabinose
11	A, B, C	0.92 ^#^	193.0354	193.0344	−5.16	C_6_H_10_O_7_	MS^2^[193]: 103.0023(100), 59.0124(29), 85.0280(16), 193.0708(10)	β-D-Glucopyranuronic acid
12	A, B, C	0.93 ^#^	133.0142	133.0130	−9.60	C_4_H_6_O_5_	MS^2^[133]: 115.0023(100), 71.0124(44), 133.0130(32)	Malic acid
13	A, B, C	0.93 ^#^	177.0405	177.0395	−5.60	C_6_H_10_O_6_	MS^2^[177]: 59.0124(100), 129.0181(42), 99.0074(34), 89.0230(32), 177.0397(31)	δ-Gluconic acidδ-lactone
14	A, B, C	0.94 ^#^	191.0197	191.0188	−5.01	C_6_H_8_O_7_	MS^2^[191]: 111.0074(100), 87.0073(35), 85.0280(28), 191.0188(11)	Citric acid isomer
15	A, B, C	1.17 ^#^	191.0197	191.0188	−5.01	C_6_H_8_O_7_	MS^2^[191]: 111.0074(100), 87.0073(34), 85.0281(23), 191.0188(10)	Citric acid
16	A, B, C	1.17 ^#^	147.0299	147.0287	−8.35	C_5_H_8_O_5_	MS^2^[147]: 129.0180(100), 85.0280(28), 103.0386(20), 147.0287(18), 101.0230(17)	α-Hydroxyglutaric acid
17	A, B, C	1.17 ^#^	243.0623	243.0617	−2.35	C_9_H_12_N_2_O_6_	MS^2^[243]: 110.0234(100), 128.0340(24), 200.0556(21), 152.0342(16), 140.0341(11)	Uridine
18	A, B, C	1.17	167.0211	167.0199	−6.79	C_5_H_4_N_4_O_3_	MS^2^[167]: 167.0200(100), 124.0140(38)	Uric acid
19	A, B, C	1.31 ^#^	161.0455	161.0444	−7.37	C_6_H_10_O_5_	MS^2^[161]: 99.0437(100), 57.0332(63), 101.0230(33), 161.0444(29), 59.0124(22)	3-Hydroxy-3-methylglutaric acid
20	A, B, C	1.42 ^#^	145.0506	145.0495	−8.02	C_6_H_10_O_4_	MS^2^[145]: 145.0494(100), 101.0594(50)	3-Methylglutaric acid
21	A, B, C	1.66 ^#^	169.0142	169.0133	−5.49	C_7_H_6_O_5_	MS^2^[169]: 125.0231(100)	Gallic acid
22	A, B, C	1.92	164.0717	164.0707	−6.23	C_9_H_11_NO_2_	MS^2^[164]: 147.0440(100), 164.0706(59), 72.0077(28), 96.9587(13)	Phenylalanine
23	A, B, C	1.96 ^#^	137.0244	137.0232	−8.74	C_7_H_6_O_3_	MS^2^[137]: 93.0331(100), 137.0231(37)	Salicylic acid
24	A, B, C	2.14 ^#^	218.1034	218.1028	−2.60	C_9_H_17_NO_5_	MS^2^[218]: 88.0389(100), 146.0810(50), 218.1027(13), 71.0124(9)	Pantothenic acid
25	A, B, C	2.28 ^#^	197.0455	197.0447	−4.40	C_9_H_10_O_5_	MS^2^[167]: 72.9917(100), 135.0439(67), 179.0340(58), 123.0439(50), 197.0445(11)	Danshensu
26	A, B, C	2.28 ^#^	417.0827	417.0800	−6.62	C_20_H_18_O_10_	MS^2^[417]: 219.0268(100), 197.0445(21), 179.0339(5)	Salvianolic acid D
27	A, B, C	2.35 ^#^	167.0350	167.0339	−6.36	C_8_H_8_O_4_	MS^2^[167]: 167.0339(100), 123.0439(87)	Vanillic acid
28	A, B, C	2.81 ^#^	153.0193	153.0183	−3.10	C_7_H_6_O_4_	MS^2^[153]: 109.0281(100), 153.0182(30), 126.0911(19)	Protocatechuic acid
29	B, C	2.96 ^#^	158.0823	158.0811	−7.38	C_7_H_13_NO_3_	MS^2^[158]: 116.0704(100), 158.0811(21)	N-Acetylvaline
30	A, B, C	3.33 ^#^	181.0506	181.0497	−5.43	C_9_H_10_O_4_	MS^2^[181]: 163.0389(100), 181.0496(68), 135.0439(54), 137.0231(42), 119.0489(34)	Ethyl 3,4-dihydroxybenzoate
31	A, B, C	3.35 ^#^	167.0350	167.0339	−6.24	C_8_H_8_O_4_	MS^2^[167]: 123.0438(100), 167.0339(10)	Isovanillic acid
32	A, B, C	3.46 ^#^	203.0826	203.0818	−3.85	C_11_H_12_N_2_O_2_	MS^2^[203]: 116.0492(100), 203.0816(70), 74.0233(36), 72.0077(32), 159.0916(31), 142.0650(29)	Tryptophan
33	A, B, C	3.66 ^#^	175.0612	175.0601	−6.16	C_7_H_12_O_5_	MS^2^[175]: 146.9600(100), 115.0387(80), 175.0601(69), 113.0594(32), 85.0644(31)	2-Isopropylmalic acid
34	A, B, C	3.77 ^#^	161.0244	161.0231	−8.25	C_9_H_6_O_3_	MS^2^[161]: 161.0233(100), 133.0282(66)	7-Hydroxycoumarin
35	A, B, C	3.80 ^#^	153.0193	153.0182	−3.82	C_7_H_6_O_4_	MS^2^[153]: 109.0282(100), 153.0181(57)	Gentisic acid
36	A, B, C	4.16 ^#^	188.0353	188.0345	−4.51	C_10_H_7_NO_3_	MS^2^[188]: 144.0443(100), 188.0343(4)	Kynurenic acid
37	C	4.51 ^#^	465.1038	465.1038	−0.20	C_21_H_22_O_12_	MS^2^[465]: 285.0403(100), 125.0232(85), 275.0563(53), 177.0189(26), 151.0033(21), 303.0518(19)	Taxifolin-glucoside
38	B, C	4.62 *^#^	289.0718	289.0721	1.00	C_15_H_14_O_6_	MS^2^[289]: 245.0816(100), 289.0721(94), 125.0233(62), 109.0283(58), 179.0340(48), 151.0390(30), 161.0594(20)	Catechin/Catechin hydrate
39	A, B, C	4.78 ^#^	163.0401	163.0390	−6.61	C_9_H_8_O_3_	MS^2^[163]: 163.0389(100), 120.0522(32)	3-Coumaric acid
40	A, B, C	5.02 ^#^	163.0401	163.0390	−6.74	C_9_H_8_O_3_	MS^2^[163]: 119.0489(100), 163.0390(16)	p-Coumaric acid
41	A, C	5.08 ^#^	465.1038	465.1037	−0.26	C_21_H_22_O_12_	MS^2^[465]: 285.0403(100), 125.0232(39), 275.0558(12), 177.0183(14), 151.0033(21), 303.0507(8)	Taxifolin-glucoside isomer
42	B, C	5.17 ^#^	172.0979	172.0969	−5.85	C_8_H_15_NO_3_	MS^2^[172]: 130.0861(100), 172.0969(16), 128.1068(2)	N-Acetyl-L-leucine
43	A, B, C	5.22	179.0350	179.0340	−5.49	C_9_H_8_O_4_	MS^2^[179]: 135.0439(100), 179.0340(29)	Caffeic acid
44	B, C	5.33 ^#^	151.0401	151.0390	−7.14	C_8_H_8_O_3_	MS^2^[151]: 107.0489(100), 151.0387(5)	2-Hydroxyphenylacetic acid
45	A, B, C	5.41 ^#^	193.0506	193.0497	−4.68	C_10_H_10_O_4_	MS^2^[193]: 134.0361(100), 149.0596(34), 193.0499(13)	Ferulic acid
46	A, B, C	5.94 ^#^	375.1310	375.1307	−0.96	C_17_H_20_N_4_O_6_	MS^2^[375]: 255.0884(100), 212.0821(18), 151.0388(18), 161.0234(14)	Riboflavin
47	C	6.01 ^#^	449.1089	449.1090	0.17	C_21_H_22_O_11_	MS^2^[449]: 259.0608(100), 59.0124(94), 269.0455(78), 125.0233(37), 287.0564(32), 178.9974(18)	Eriodictyol-glucoside
48	A, B, C	6.01 ^#^	206.0823	206.0817	−3.00	C_11_H_13_NO_3_	MS^2^[206]: 164.0706(100), 147.0440(28), 58.0285(24), 206.0814(21), 70.0285(14)	N-Acetyl-L-phenylalanine
49	A, B, C	6.16 ^#^	167.0350	167.0340	−5.88	C_8_H_8_O_4_	MS^2^[167]: 167.0339(100), 152.0103(19)	4-Methoxysalicylic acid
50	A, B, C	6.49 ^#^	173.0819	173.0809	−5.79	C_8_H_14_O_4_	MS^2^[173]: 111.0802(100), 173.0809(55), 129.0908(6), 112.0835(5)	Suberic acid
51	A, B, C	6.54 ^#^	537.1038	537.1036	0.52	C_27_H_22_O_12_	MS^2^[537]: 339.0504(100), 229.0137(64), 295.0609(56), 197.0446(31), 135.0439(25), 179.0338(14)	Salvianolic acid U
52	A, B, C	6.54 ^#^	163.0401	163.0390	−6.61	C_9_H_8_O_3_	MS^2^[163]: 119.0490(100), 163.0389(14), 120.0522(6)	4-Coumaric acid isomer
53	A, B, C	6.61 ^#^	313.0718	313.0715	−0.74	C_17_H_14_O_6_	MS^2^[313]: 109.0281(100), 147.0439(38), 159.0440(27), 269.0816(14)	Salvianolic acid F
54	A, B, C	6.67 ^#^	174.0561	174.0550	−5.82	C_10_H_9_NO_2_	MS^2^[174]: 146.9600(100), 174.0550(67), 130.0650(35)	Indole-3-acetic acid
55	A, B, C	6.71 ^#^	537.1038	537.1036	0.52	C_27_H_22_O_12_	MS^2^[537]: 197.0446(100), 135.0439(80), 339.0505(68), 229.0137(64), 295.0609(63), 179.0340(42)	Salvianolic acid T
56	A, B, C	6.91 ^#^	537.1038	537.1033	−0.97	C_27_H_22_O_12_	MS^2^[537]: 197.0447(100), 135.0439(71), 339.0506(64), 295.0609(58), 229.0137(47), 179.0340(41)	Salvianolic acid J
57	B, C	6.91 ^#^	313.0718	313.0711	−2.09	C_17_H_14_O_6_	MS^2^[313]: 269.0818(100), 313.0716(46), 203.0341(44), 159.0443(31), 109.0281(22)	Salvianolic acid F isomer
58	A, B, C	6.91 ^#^	493.1140	493.1135	−1.08	C_26_H_22_O_10_	MS^2^[493]: 197.0446(100), 135.0439(63), 295.0609(34), 179.0339(33), 72.9917(24), 269.0818(21)	Salvianolic acid A isomer
59	C	7.03 ^#^	433.1140	433.1140	−0.17	C_21_H_22_O_10_	MS^2^[433]: 271.0609(100), 151.0030(30), 98.9477(74), 119.0489(11)	Naringenin-glucoside
60	A, B, C	7.13 ^#^	193.0506	193.0497	−4.83	C_10_H_10_O_4_	MS^2^[193]: 134.0361(100), 193.0496(13), 149.0596(7)	Isoferulic acid
61	C	7.22 ^#^	449.1089	449.1087	−0.53	C_21_H_22_O_11_	MS^2^[449]: 151.0025(100), 287.0558(65), 135.0439(46), 98.9477(41), 96.9587(12)	Eriodictyol hexoside 1
62	C	7.30 ^#^	433.1140	433.1134	−1.44	C_21_H_22_O_10_	MS^2^[433]: 271.0610(100), 151.0025(33), 98.9476(10), 119.0492(6)	Naringenin-glucoside isomer
63	A, B, C	7.50 *^#^	609.1461	609.1456	−0.79	C_27_H_30_O_16_	MS^2^[609]: 300.0273(100), 301.0345(57)	Rutin
64	A, B, C	7.57 ^#^	137.0244	137.0231	−9.40	C_7_H6O3	MS^2^[137]: 93.0332(100), 137.0232(52)	Salicylic acid isomer
65	B, C	7.60 ^#^	715.1305	715.1303	−0.30	C_36_H_28_O_16_	MS^2^[715]: 197.0446(100), 151.0390(54), 135.0437(40), 177.0182(39), 179.0339(35)	DidehydioSalvianolic acid B
66	C	7.77 *^#^	463.0882	463.0881	−0.28	C_21_H_20_O_12_	MS^2^[463]: 300.0272(100), 301.0346(56), 151.0025(4), 178.9980(2)	Isoquercitrin
67	A, B, C	7.81 ^#^	163.0401	163.0390	−6.74	C_9_H_8_O_3_	MS^2^[163]: 163.0389(100), 137.0596(91), 119.0489(46), 162.8380(17)	4-Coumaric acid
68	A, B, C	7.88 ^#^	551.1195	551.1198	0.46	C_28_H_24_O_12_	MS^2^[551]: 197.0447(100), 135.0440(74), 59.0124(62), 353.0659(50), 179.0341(45), 309.0770(41)	Monomethyl lithospermate isomer
69	A, B, C	7.92 ^#^	717.1461	717.1456	−0.75	C_36_H_30_O_16_	MS^2^[717]: 339.0505(100), 321.0760(40), 295.0611(20), 197.0449(26), 135.0440(16), 179.03469(6)	Salvianolic acid B isomer
70	A, B, C	8.02 ^#^	537.1038	537.1034	−0.86	C_27_H_22_O_12_	MS^2^[537]: 295.0609(100), 339.0504(44), 109.0282(41), 185.0233(31), 277.0504(13)	Salvianolic acid isomer
71	A, B, C	8.19 ^#^	357.0616	357.0611	−1.46	C_18_H_14_O_8_	MS^2^[357]: 135.0439(100), 229.0135(31), 197.0448(29), 179.0341(23), 109.0280(17)	Salvianolic acid H
72	B, C	8.31 ^#^	277.1445	277.1443	−0.99	C_16_H_22_O_4_	MS^2^[277]: 259.1336(100), 247.1335(88), 174.0675(82), 121.0282(76), 241.1230(47)	De-O-Methyllasiodiplodin
73	A, B, C	8.37 *^#^	593.1512	593.1509	−0.43	C_27_H_30_O_15_	MS^2^[593]: 285.0401(100), 284.0324(49)	Nicotiflorin
74	A, B, C	8.40 ^#^	187.0976	187.0966	−5.25	C_9_H_16_O_4_	MS^2^[187]: 125.0959(100), 187.0966(50), 97.0645(4), 169.0859(3)	Azelaic acid
75	A, B, C	8.50 ^#^	551.1195	551.1201	1.13	C_28_H_24_O_12_	MS^2^[551]: 327.0827(100), 135.0439(37), 197.0446(33), 217.0499(24), 229.0137(22), 353.0664(22)	Monomethyl lithospermate
76	A, B	8.65 *^#^	609.1825	609.1829	1.61	C_28_H_34_O_15_	MS^2^[609]: 301.0714(100), 302.0744(8)	Hesperidin
77	A, B, C	8.84 *	359.0772	359.0769	−0.95	C_18_H_16_O_8_	MS^2^[359]: 161.0233(100), 197.0446(37), 179.0339(16), 72.9917(11), 135.0440(6)	Rosmarinic acid
78	A, B, C	8.88 ^#^	731.1618	731.1613	−0.60	C_37_H_32_O_16_	MS^2^[731]: 109.0282(100), 335.0921(89), 353.0670(70), 197.0446(61), 489.1185(45)	9″-Methyl salvianolate B isomer
79	A, B, C	9.00 ^#^	731.1618	731.1620	0.32	C_37_H_32_O_16_	MS^2^[731]: 367.0821(100), 353.0667(97), 109.0282(74), 197.0446(58), 335.0924(53), 489.1198(35)	9″-Methyl salvianolate B
80	A, B, C	9.22 *	537.1038	537.1036	−0.51	C_27_H_22_O_12_	MS^2^[537]: 339.0511(100), 197.0447(82), 135.0440(74), 295.0613(66), 179.0338(25)	Lithospermic acid
81	A, B, C	9.22 ^#^	493.1140	493.1138	−0.45	C_26_H_22_O_10_	MS^2^[493]: 185.0235(100), 109.0281(94), 295.0608(77), 203.0343(21), 159.0440(20), 135.0439(19), 197.0448(17), 179.0343(12)	Salvianolic acid A
82	C	9.68 ^#^	493.1140	493.1140	−0.09	C_26_H_22_O_10_	MS^2^[493]: 197.0446(100), 135.0439(82), 295.0608(55), 179.0338(38), 185.0234(33), 109.0281(33), 269.0817(30)	Salvianolic acid A isomer
83	C	10.19 *	717.1461	717.1460	−0.15	C_36_H_30_O_16_	MS^2^[717]: 321.0401(100), 339.0507(29), 295.0609(16), 185.0237(10), 197.0444(3), 135.0438(2), 179.0339(2)	Salvianolic acid B
84	A, B, C	10.33 ^#^	357.0616	357.0616	−0.12	C_18_H_14_O_8_	MS^2^[357]: 135.0439(100), 337.0353(56), 72.9917(27), 179.0339(26), 197.0446(25), 321.0403(18)	Salvianolic acid I
85	A, B, C	10.66 ^#^	731.1618	731.1625	1.07	C_37_H_32_O_16_	MS^2^[731]: 229.0136(100), 339.0506(76), 313.0716(59), 203.0340(47), 267.0659(32)	9″-Methyl salvianolate B isomer
86	A, B, C	11.04 ^#^	277.1445	277.1441	−1.56	C_16_H_22_O_4_	MS^2^[277]: 277.1443(100), 233.1541(74), 203.1433(11)	Dibutylphthalate
87	B, C	11.16 ^#^	373.0929	373.0926	−0.73	C_19_H_18_O_8_	MS^2^[373]: 135.0439(100), 175.0391(65), 197.0447(57), 179.0340(27), 72.9917(23), 161.0235(15)	Methyl rosmarinate
88	A, B, C	11.20 ^#^	201.1132	201.1125	−3.79	C_10_H_18_O_4_	MS^2^[201]: 139.1116(100), 201.1123(92), 183.1017(47)	3-tert-Butyladipic acid
89	A, B, C	11.65 *	491.0984	491.0980	−0.84	C_26_H_20_O_10_	MS^2^[491]: 311.0559(100), 135.0439(35), 197.0447(4), 179.0341(2)	Salvianolic acid C
90	C	11.87 ^#^	551.1195	551.1193	−0.44	C_28_H_24_O_12_	MS^2^[551]: 321.0400(100), 231.0292(22), 109.0281(21), 293.0455(17), 135.0440(11), 197.0447(8), 179.0337(4)	Monomethyl lithospermate isomer
91	B, C	11.92 ^#^	731.1618	731.1627	1.32	C_37_H_32_O_16_	MS^2^[731]: 335.0560(100), 353.0666(60), 309.0762(38), 135.0439(33), 197.0446(20)	9″-Methyl salvianolate B isomer
92	A	12.49 *^#^	301.0354	301.0353	−0.22	C_15_H_10_O_7_	MS^2^[301]: 151.0026(100), 301.0359(89), 178.9977(53), 121.0283(19)	Quercetin
93	C	13.01 ^#^	731.1618	731.1630	1.73	C_37_H_32_O_16_	MS^2^[731]: 339.0507(100), 229.0137(42), 295.0607(30), 359.0772(19)	9″-Methyl salvianolate B isomer
94	A, B, C	13.13 ^#^	363.1085	363.1086	0.03	C_18_H_20_O_8_	MS^2^[363]: 218.0214(100), 190.0262(79), 303.0872(74), 227.0343(70), 219.0251(5)	Shikonin derivative
95	C	13.25 *^#^	271.0612	271.0613	0.31	C_15_H_12_O_5_	MS^2^[271]: 151.0025(100), 271.0612(62), 119.0490(42), 227.1071(23), 107.0125(14), 93.0332(14), 177.0185(11)	Naringenin
96	A, B, C	14.54 ^#^	285.0405	285.0408	1.30	C_15_H_10_O_6_	MS^2^[285]: 285.0406(100), 227.0711(16), 241.0508(11), 215.0701(11)	Kaempferol
97	A, B, C	14.56	253.0870	253.0869	−0.67	C_16_H_14_O_3_	MS^2^[253]: 237.0551(100), 238.0615(31), 270.0533(20), 253.0505(14)	Rhizonone
98	A, B, C	14.57	315.1238	315.1239	0.43	C_18_H_20_O_5_	MS^2^[315]: 241.0865(100), 256.1102(65), 300.1002(27), 271.0973(22), 285.0762(11)	Ethylshikonin
99	A, B, C	14.61 ^#^	299.0561	299.0561	0.03	C_16_H_12_O_6_	MS^2^[301]: 284.0323(100), 255.0293(16), 299.0560(12), 285.0359(10)	Hispidulin
100	C	15.35 ^#^	583.2562	583.2564	0.24	C_33_H_36_N_4_O_6_	MS^2^[583]: 285.1242(100), 297.1239(15), 241.1341(9), 213.1030(3)	Bilirubin
101	A, B, C	15.35 ^#^	187.1340	187.1331	−4.53	C_10_H_20_O_3_	MS^2^[187]: 59.0124(100), 125.0960(21), 141.8672(14), 187.1324(14)	3-Hydroxydecanoic acid
102	A, B, C	15.74 ^#^	371.1500	371.1497	−0.87	C_21_H_24_O_6_	MS^2^[371]: 271.0970(100), 253.0863(52), 99.0439(49), 241.0868(40), 225.0916(38)	Valerylshikonin isomer
103	A, B, C	15.85 ^#^	403.1398	403.1398	−0.03	C_21_H_24_O_8_	MS^2^[403]: 303.0875(100), 218.0215(96), 227.0345(65), 190.0258(75), 99.0435(83)	Shikonin derivative
104	B, C	15.96	391.1762	391.1762	−0.20	C_21_H_28_O_7_	MS^2^[391]: 255.1024(100), 273.1133(92), 190.0262(18), 117.0544(17), 227.0340(14)	Hydroxyshikonofuran J
105	A, B, C	15.99 ^#^	391.1398	391.1399	0.21	C_20_H_24_O_8_	MS^2^[363]: 218.0215(100), 303.0872(98), 227.0343(87), 190.0262(82), 87.0437(71)	Shikonin derivative
106	A, B, C	16.05	269.0819	269.0819	−0.09	C_16_H_14_O_4_	MS^2^[269]: 136.0153(100), 251.0712(58), 223.0753(44), 269.0817(41), 241.0870(31)	Dehydratedshikonin
107	A, B, C	16.13	333.1344	333.1341	−0.82	C_18_H_22_O_6_	MS^2^[333]: 255.1023(100), 273.1129(66), 219.0292(15), 254.0937(2), 237.0917(1)	Hydroxyshikonofuran A
108	B, C	16.31	391.1762	391.1763	0.27	C_21_H_28_O_7_	MS^2^[391]: 255.1023(100), 273.1133(91), 117.0545(25)	Hydroxyshikonofuran K
109	C	16.36 ^#^	283.0612	283.0611	−0.45	C_16_H_12_O_5_	MS^2^[283]: 283.0608(100), 240.0423(27), 257.0451(19), 239.0373(3)	Glycitein
110	A, B, C	16.46 ^#^	333.1344	333.1343	−0.07	C_18_H_22_O_6_	MS^2^[333]: 255.1023(100), 273.1130(65), 315.1576(30), 254.0945(2), 237.0918(2)	Hydroxyshikonofuran A isomer
111	A, B, C	16.76	315.1238	315.1238	−0.06	C_18_H_20_O_5_	MS^2^[315]: 255.1023(100), 59.0125(29), 227.1077(13), 237.0922(11), 121.0281(7), 187.0390(4)	Shikonofuran A
112	A, B, C	16.76	347.1136	347.1134	−0.57	C_18_H_20_O_7_	MS^2^[347]: 241.0867(100), 287.0922(73), 269.0824(69), 59.0124(68), 227.1079(23)	Shikonin acetate
113	A, B, C	16.79 ^#^	305.1758	305.1758	1.63	C_18_H_26_O_4_	MS^2^[305]: 135.0803(100), 249.1492(62), 174.9551(46), 235.0195(26), 146.9600(26)	Octyl ferulate
114	C	16.87 *^#^	283.0612	283.0612	−0.03	C_16_H_12_O_5_	MS^2^[283]: 283.0608(100), 268.0377(67), 265.1805(10)	Baicalein
115	A, B, C	17.67 ^#^	349.1293	349.1292	−0.37	C_18_H_22_O_7_	MS^2^[349]: 255.1023(100), 227.1071(43), 237.0921(13)	Shikonofurans derivative
116	A, B, C	17.00	405.1555	405.1558	0.74	C_21_H_26_O_8_	MS^2^[405]: 303.0870(100), 218.0214(59), 190.0262(58), 227.0343(56), 245.0451(54), 101.0594(53)	Dihydrohydroxyshikonin tiglate
117	A, B, C	17.11 *	299.0561	299.0560	−0.38	C_16_H_12_O_6_	MS^2^[299]: 299.0558(100), 254.0581(27), 237.0556(25), 281.0452(22), 284.0333(13)	Kaempferide
118	A, B, C	17.14 ^#^	313.0718	313.0716	−0.46	C_17_H_14_O_6_	MS^2^[313]: 298.0482(100), 202.1159(82), 283.0243(53), 312.2260(47), 255.0294(24)	Pectolinarigenin
119	B, C	17.19	345.0980	345.0975	−1.47	C_18_H_18_O_7_	MS^2^[345]: 285.0766(100), 267.0674(32), 257.0807(25), 59.0123(20)	Lithospermidin C
120	A, B, C	18.04 *	287.0925	287.0923	−0.62	C_16_H_16_O_5_	MS^2^[287]: 218.0214(100), 219.0255(9), 190.0261(2)	Shikonin
121	B, C	18.15	433.1868	433.1871	0.65	C_23_H_30_O_8_	MS^2^[433]: 255.1023(100), 273.1131(84), 273.9557(16), 59.0126(5)	Hydroxyshikonofuran F
122	A, B, C	18.17	361.1657	361.1655	−0.54	C_20_H_26_O_6_	MS^2^[361]: 255.1022(100), 273.1129(73), 259.0607(15), 87.0439(4)	Hydroxyshikonofuran G
123	B, C	18.45	433.1868	433.1866	−0.56	C_23_H_30_O_8_	MS^2^[433]: 255.1023(100), 273.1131(81), 59.0123(6), 237.0913(3)	Hydroxyshikonofuran H
124	A, B, C	18.47	361.1657	361.1658	0.39	C_20_H_26_O_6_	MS^2^[361]: 255.1022(100), 273.1128(72), 218.0214(10), 87.0438(3)	Hydroxyshikonofuran D
125	A, B, C	18.57 ^#^	269.0455	269.0454	3.57	C_15_H_10_O_5_	MS^2^[301]: 269.0454(100), 149.0229(1)	Apigenin
126	A, B, C	18.65 ^#^	345.0980	345.0975	−1.47	C_18_H_18_O_7_	MS^2^[345]: 285.0766(100), 267.0659(14), 257.0817(8), 59.0124(4), 239.0709(1)	Lithospermidin C isomer
127	A, B, C	18.83	343.1551	343.1551	−0.03	C_20_H_24_O_5_	MS^2^[343]: 283.0974(100), 266.0824(14), 255.1023(35), 87.0438(16), 227.1078(3)	Shikonofuran D
128	A, B, C	18.89	343.1187	343.1184	−0.80	C_19_H_20_O_6_	MS^2^[343]: 283.0974(100), 255.1023(35), 87.0438(16), 266.0824(14)	1-Methoxyacetylshikonin
129	A, B, C	18.92	373.1657	373.1658	0.37	C_21_H_26_O_6_	MS^2^[373]: 255.1022(100), 273.1131(61), 174.9551(35)	Hydroxyshikonofuran I
130	C	18.99 ^#^	357.2071	357.2071	−0.04	C_22_H_30_O_4_	MS^2^[357]: 357.2070(100), 339.1964(69), 295.2061(31), 327.1965(30), 269.0819(13)	Cannabidiolic acid
131	A, B, C	19.04 ^#^	269.0819	269.0812	−0.20	C_16_H_14_O_4_	MS^2^[269]: 269.0816(100), 254.0582(80), 149.0231(42), 133.0646(37), 210.0677(35)	Medicarpin
132	A, B, C	19.16	373.1657	373.1655	−0.36	C_21_H_26_O_6_	MS^2^[373]: 255.1025(100), 273.1130(67), 174.9550(24)	Hydroxyshikonofuran E
133	B, C	19.33	375.1813	375.1812	1.03	C_21_H_28_O_6_	MS^2^[375]: 255.1025(100), 273.1132(71)	Hydroxyshikonofuran B
134	A, B, C	19.33	343.1187	343.1185	−0.53	C_19_H_20_O_6_	MS^2^[343]: 283.0973(100), 255.1021(13)	1-methoxyacetylshikonin isomer
135	A, B, C	19.49	375.1813	375.1810	0.72	C_21_H_28_O_6_	MS^2^[375]: 255.1023(100), 273.1132(74)	Hydroxyshikonofuran L
136	A, B, C	19.52	355.1551	355.1546	−1.49	C_21_H_24_O_5_	MS^2^[355]: 255.1025(100), 355.3217(70), 99.0438(58), 218.0218(30), 227.1070(15), 237.0919(4)	Shikonofuran E
137	B, C	19.67	375.1813	375.1784	−7.74	C_21_H_28_O_6_	MS^2^[375]: 255.1024(100), 273.1131(69), 101.0594(3)	Hydroxyshikonofuran C
138	A, B, C	19.74	359.1136	359.1139	0.82	C_19_H_20_O_7_	MS^2^[359]: 299.0921(100), 284.0687(57), 359.1137(11), 161.0234(9), 271.0973(7)	1/4-methoxylithospermidin C
139	A, B, C	19.80	425.1242	425.1245	1.90	C_23_H_22_O_8_	MS^2^[425]: 321.1494(100), 178.9977(59), 227.1074(58), 271.0969(27), 363.1226(21), 245.1178(16), 345.1130(12)	Unknown
140	A, B, C	19.80	375.1813	375.1811	−0.52	C_21_H_28_O_6_	MS^2^[375]: 255.1023(100), 273.1131(72), 101.0594(3)	Hydroxyshikonofuran M
141	A, B	20.06	357.1707	357.1705	−0.61	C_21_H_26_O_5_	MS^2^[357]: 255.1023(100), 101.0594(47), 297.1130(18), 227.1069(9), 121.0281(7), 237.0916(6)	Shikonofuran B
142	A, B, C	20.15	357.1707	357.1704	−0.95	C_21_H_26_O_5_	MS^2^[357]: 255.1023(100), 101.0594(45), 227.1068(10), 237.0921(8), 121.0283(8), 172.0517(2), 143.0497(2)	Shikonofuran C
143	A, B, C	20.16	371.1500	371.1497	−0.79	C_21_H_24_O_6_	MS^2^[371]: 269.0817(100), 241.0866(16), 251.0706(7)	α,α-dimethylpropionylshikonin
144	A, B, C	20.50	387.1449	387.1448	−0.30	C_21_H_24_O_7_	MS^2^[387]: 117.0544(100), 269.0813(31), 251.0711(31), 59.0123(21), 241.0867(13)	β-hydroxyisovalerylshikonin
145	A, B, C	20.94	401.1606	401.1605	1.16	C_22_H_26_O_7_	MS^2^[401]: 299.0923(100), 255.1027(57), 121.0284(15), 313.0705(10), 237.0908(10)	1-Methoxy-β-hydroxyisovalerylshikonin
146	A, B, C	21.33	387.1449	387.1448	−0.23	C_21_H_24_O_7_	MS^2^[387]: 101.0594(100), 189.0184(76), 285.0765(60), 217.0135(49), 257.0814(28), 267.0659(19)	Lithospermidin A
147	B, C	21.69	459.1661	459.1664	0.71	C_24_H_28_O_9_	MS^2^[459]: 299.0916(100), 59.0124(58), 271.0966(36), 281.0820(21)	1/4-methoxylithospermidin H
148	B, C	21.70	445.1504	445.1503	−0.18	C_23_H_26_O_9_	MS^2^[445]: 285.0766(100), 257.0819(9), 59.0124(8), 267.0657(7)	Lithospermidin D
149	A, B	21.76 ^#^	271.0976	271.0974	−0.53	C_16_H_16_O_4_	MS^2^[271]: 253.0867(100), 271.0968(73), 203.0342(58), 256.0739(44), 238.0630(39)	Deoxyshikonin isomer
150	A, B, C	21.89 *	329.1031	329.1030	−0.19	C_18_H_18_O_6_	MS^2^[329]: 269.0818(100), 251.0710(81), 241.0868(48), 59.0124(47)	Acetylshikonin
151	A, B, C	22.80	387.1449	387.1449	0.01	C_21_H_24_O_7_	MS^2^[387]: 299.0923(100), 270.0893(94), 285.0756(68), 271.0954(66), 87.0438(63), 253.0864(24)	1/4-methoxylithospermidin I
152	A, B, C	22.84	373.1293	373.1293	0.15	C_20_H_22_O_7_	MS^2^[373]: 285.0768(100), 267.0663(10), 257.0817(9), 174.9552(8), 87.0438(6)	Lithospermidin E
153	B, C	23.03 ^#^	445.1504	445.1497	−1.61	C_23_H_26_O_9_	MS^2^[445]: 285.0768(100), 257.0817(49), 59.0123(18), 267.0655(7)	Lithospermidin D isomer
154	A, B, C	23.13	387.1449	387.1447	−0.56	C_21_H_24_O_7_	MS^2^[387]: 299.0903(100), 285.0767(78), 270.0897(74), 87.0438(58), 271.0974(50)	1/4-methoxylithospermidin E
155	A, B, C	24.03	285.0768	285.0767	−0.66	C_16_H_14_O_5_	MS^2^[285]: 285.0766(100), 267.0659(55), 227.0344(50), 73.0281(8), 257.0817(6), 239.0708(2)	Sakuranetin
156	B, C	24.23 ^#^	445.1504	445.1504	−0.11	C_23_H_26_O_9_	MS^2^[445]: 285.0771(100), 257.0809(93), 59.0124(35)	Lithospermidin D isomer
157	A, B, C	24.57	399.1449	399.1445	−1.07	C_22_H_24_O_7_	MS^2^[399]: 270.0891(100), 299.0921(98), 271.0978(68), 99.0437(56), 281.0811(21)	1/4-methoxylithospermidin J
158	A, B, C	24.63	385.1293	385.1292	−0.33	C_21_H_22_O_7_	MS^2^[385]: 285.0767(100), 257.0817(11), 99.0438(9), 267.0651(8)	Lithospermidin F
159	A, B, C	24.81	399.1449	399.1445	−1.07	C_22_H_24_O_7_	MS^2^[399]: 299.0921(100), 271.0957(63), 99.0438(53), 281.0811(25)	1/4-methoxylithospermidin F
160	A, B, C	24.99	343.1187	343.1193	1.69	C_19_H_20_O_6_	MS^2^[343]: 57.0332(100), 343.2271(79), 269.0806(55), 251.0713(51), 285.1859(50), 73.0281(43), 241.0872(33)	Propionylshikonin
161	A, B, C	25.48	401.1606	401.1603	−0.67	C_22_H_26_O_7_	MS^2^[401]: 299.0919(100), 270.0900(92), 101.0594(61), 271.0976(53), 281.0826(21), 253.0871(17)	1/4-methoxylithospermidin A
162	A, B, C	25.60	387.1449	387.1448	−0.30	C_21_H_24_O_7_	MS^2^[387]: 285.0766(100), 257.0816(9), 101.0594(9), 267.0658(8)	Lithospermidin B
163	B, C	26.29	459.1661	459.1654	−1.41	C_24_H_28_O_9_	MS^2^[459]: 299.0922(100), 271.0971(10), 59.0124(9)	1/4-methoxylithospermidin D
164	B	26.38 ^#^	445.1504	445.1500	−0.94	C_23_H_26_O_9_	MS^2^[445]: 285.0768(100), 257.0815(54), 59.0125(21), 267.0662(6)	Lithospermidin D isomer
165	B	26.38 ^#^	271.0976	271.0974	−0.86	C_16_H_16_O_4_	MS^2^[271]: 253.0867(100), 271.0966(71), 203.0341(57), 256.0736(48), 238.0629(39)	Deoxyshikonin isomer
166	A, B, C	26.60	429.1555	429.1552	−0.59	C_23_H_26_O_8_	MS^2^[429]: 269.0822(100), 251.0710(65), 59.0125(65), 241.0866(35)	5-acetoxy-valerylshikonin
167	A, B	28.04 ^#^	271.0976	271.0974	−0.53	C_16_H_16_O_4_	MS^2^[271]: 253.0866(100), 271.0967(66), 203.0341(54), 256.0738(45), 238.0629(42)	Deoxyshikonin isomer
168	B, C	28.07	429.1555	429.1551	−0.87	C_23_H_26_O_8_	MS^2^[429]: 269.0822(100), 251.0706(63), 59.0124(61), 241.0871(37)	β-acetoxyisovalerylshikonin
169	A, B, C	28.08 ^#^	425.1242	425.1227	−2.13	C_23_H_22_O_8_	MS^2^[425]: 269.0818(100), 251.0710(27), 87.0437(17), 241.0966(10)	Shikonin derivative
170	A, B, C	28.16	357.1344	357.1342	−0.40	C_20_H_22_O_6_	MS^2^[357]: 269.0819(100), 251.0713(70), 87.0438(63), 241.0863(50), 223.0820(4)	Butyrylshikonin
171	A, B, C	29.19	357.1344	357.1342	−0.40	C_20_H_22_O_6_	MS^2^[357]: 269.0819(100), 251.0707(66), 87.0437(72), 241.0866(43)	Isobutyrylshikonin
172	A, B, C	29.53	399.1449	339.1999	9.71	C_22_H_24_O_7_	MS^2^[399]: 299.0923(100), 99.0439(11), 281.0812(10), 271.0970(9)	1/4-Methoxylithospermidin L
173	A, B, C	30.11 ^#^	271.0976	271.0974	−0.64	C_16_H_16_O_4_	MS^2^[271]: 253.0867(100), 271.0967(74), 203.0341(54), 256.0736(45), 238.0631(41)	Deoxyshikonin isomer
174	A, B, C	30.19 *	369.1344	369.1342	−0.39	C_21_H_22_O_6_	MS^2^[369]: 269.0818(100), 251.0710(71), 99.0438(68), 270.0888(57), 241.0867(49)	β,β-dimethylacrylshikonin
175	A, B, C	30.44	401.1606	401.1603	−0.59	C_22_H_26_O_7_	MS^2^[401]: 299.0924(100), 121.0283(15), 271.0959(12), 101.0592(11), 281.0879(10)	1/4-methoxylithospermidin B
176	A, B, C	30.64	599.1923	599.1926	1.52	C_34_H_32_O_10_	MS^2^[599]: 426.1099(100), 412.0935(42), 102.9554(26), 132.4304(21), 116.5537(21), 59.0124(21)	7-(11′-Deoxyalkannin)-Acetylshikonin
177	A	30.72	369.1344	369.1340	−1.07	C_21_H_22_O_6_	MS^2^[369]: 269.0817(100), 251.0709(68), 99.0438(61), 241.0864(52)	α-methylene-butenoylshikonin
178	A, B, C	30.89 ^#^	271.0976	271.0974	−0.53	C_16_H_16_O_4_	MS^2^[271]: 253.0868(100), 271.0968(77), 203.0343(64), 256.0738(47), 238.0630(40)	Deoxyshikonin isomer
179	A, B, C	30.98	371.1500	371.1497	−0.95	C_21_H_24_O_6_	MS^2^[371]: 269.0818(100), 251.0710(66), 101.0594(65), 241.0865(43)	α-methylbutyrylshikonin
180	A, B, C	31.62	555.1661	555.1657	−0.63	C_32_H_28_O_9_	MS^2^[555]: 486.0952(100), 555.1646(11)	Shikometabolin B
181	A, B, C	31.78	599.1923	599.1936	3.14	C_34_H_32_O_10_	MS^2^[599]: 412.0944(100), 426.1105(77)	7-(11′-Deoxyalkannin)-Acetylalkannin
182	A	32.05	369.1344	369.1343	−0.14	C_21_H_22_O_6_	MS^2^[369]: 269.0816(100), 251.0705(48), 99.0439(43), 241.0871(37)	Tigloylshikonin
183	A, B, C	33.05	371.1500	371.1497	−0.95	C_21_H_24_O_6_	MS^2^[371]: 269.0819(100), 101.0594(71), 251.0710(64), 241.0866(47)	Isovalerylshikonin
184	A, B, C	34.64 ^#^	271.0976	271.0973	−1.08	C_16_H_16_O_4_	MS^2^[271]: 253.0866(100), 271.0974(66), 203.0341(58), 256.0735(45), 238.0630(37)	Deoxyshikonin isomer
185	A	35.15	369.1344	369.1342	−0.47	C_21_H_22_O_6_	MS^2^[369]: 269.0829(100), 251.0721(46), 99.0443(54), 241.0867(40)	Angeloylshikonin
186	A, B, C	35.84	627.2236	627.2235	0.69	C_36_H_36_O_10_	MS^2^[627]: 426.1100(100), 412.0944(80), 87.0437(45), 495.1801(24), 349.1006(12), 290.5349(12)	7-(11′-Deoxyalkannin)-Isobutyrylshikonin
187	A, B, C	38.59	639.2236	639.2231	0.19	C_37_H_36_O_10_	MS^2^[639]: 537.1556(100), 639.2231(5), 509.1626(4), 519.1473(2), 101.0590(2)	7-(11′-Deoxyalkannin)-β,β-dimethylacrylshikonin
188	A, B, C	38.89	639.2236	639.2232	0.30	C_37_H_36_O_10_	MS^2^[639]: 537.1553(100), 639.2230(4), 519.1443(2), 101.0589(1)	7-(11′-Deoxyalkannin)-β,β-dimethylacrylalkannin

* Identified by comparison with standards. ^#^ First reported in *Arnebiae Radix*.

## Data Availability

Data will be provided upon request.

## Sample Availability

Samples of the compounds are available from the authors.
